# Synthetic Cooling Agents in Australian-Marketed E-cigarette Refill Liquids and Disposable E-cigarettes: Trends Follow the U.S. Market

**DOI:** 10.1093/ntr/ntad120

**Published:** 2023-07-14

**Authors:** Caitlin Jenkins, Jody Morgan, Celine Kelso

**Affiliations:** Molecular Horizons, University of Wollongong, Wollongong, NSW, Australia; School of Chemistry and Molecular Bioscience, University of Wollongong, Wollongong, NSW, Australia; Molecular Horizons, University of Wollongong, Wollongong, NSW, Australia; School of Chemistry and Molecular Bioscience, University of Wollongong, Wollongong, NSW, Australia; Molecular Horizons, University of Wollongong, Wollongong, NSW, Australia; School of Chemistry and Molecular Bioscience, University of Wollongong, Wollongong, NSW, Australia

## Abstract

**Introduction:**

E-cigarettes are becoming increasingly popular in Australia, especially amongst the younger population. The synthetic cooling molecules WS-3 and WS-23 have been identified in e-cigarette products from the United States and Europe. The extent of inclusion of these synthetic coolants in Australian e-liquids is unknown, particularly in newer disposable e-cigarettes.

**Aims and Methods:**

E-cigarettes and e-liquids were purchased within Australia and anonymously donated by Australian users. Nicotine, WS-3, WS-23, and menthol were quantified in the e-liquids using gas chromatography-mass spectrometry (GC-MS).

**Results:**

WS-23 and nicotine were detected in all of the disposable e-cigarettes with WS-23 often present in high concentrations. There was no correlation between cooling terms in the flavor name and the inclusion of cooling agents. Only three bottled e-liquids were found to contain WS-23 while none contained WS-3 above the limit of detection.

**Conclusions:**

Synthetic coolants were a common addition in disposable e-cigarettes while rarely added to e-liquid bottle refills. Their inclusion in these products is reflective of trends observed in United States and European e-cigarette products.

**Implications:**

The increase in synthetic cooling agents as components of e-liquids, particularly disposable e-cigarette devices, has been observed within Australian samples across a range of brands and flavors. WS-23 was present in every disposable e-cigarette analyzed in this study, often in relatively high concentrations. Its inhalational toxicology should be considered when evaluating the safety of these products.

## Introduction

Electronic cigarettes (e-cigarettes) are increasingly popular devices that allow users to inhale a vaporized liquid (e-liquid), often containing nicotine and offering potential as a smoking cessation device.^1^ Currently, e-cigarettes and e-liquids containing nicotine are banned in Australia for non-therapeutic use, with Australian retailers (retail shops and Australian online suppliers) only allowed to sell nicotine-free vaping products.^2^ Individuals requiring access to nicotine-containing e-liquids may import up to 3 months' supply of nicotine-containing e-liquids from overseas providers with a prescription from a medical practitioner.^2^ The Therapeutic Goods Administration’s Standard for Nicotine Vaping Products (TGO110), which commenced on October 1, 2021, sets out minimum safety and quality requirements for nicotine vaping products that are not registered in the Australian Register of Therapeutic Goods and are imported into, supplied in or exported from Australia.^3^ This includes, packaging requirements and limitations on content such as prohibition of chemicals with known inhalational toxicities. The TGO110 only applies to nicotine-containing products, with nicotine-free products being exempt. This split regulation has resulted in a wide range of unlabeled nicotine-containing e-liquid products being easily accessible on the black market or obtained over the internet.

Within Australia, 9.3% of individuals aged 18 years or older had ever used an e-cigarette in 2021^.4^ The popularity of e-cigarettes can be partially attributed to the extensive range of flavors available, including fruity, dessert, and tobacco flavors.^5^ In addition to the suite of molecules composing each of these flavors, some may also include the presence of cooling agents which produce a cold mouth-feel when inhaled.^6^ Two of these synthetic cooling agents, WS-3 (*N*-ethyl-p-menthane-3-carboxamide) and WS-23 (2-isopropyl-*N*,2,3-trimethylbutyramide), provide a cooling sensation without the minty flavor that is generally associated with menthol.^6,7^ The addition of these cooling agents may also be an attempt to reduce the throat irritation experienced by the user in products with a high concentration of nicotine,^6,8^ particularly among nicotine-naïve individuals. Recent recommendations in Australia have promoted the limitation of e-cigarette products to unflavored or tobacco-flavored only in an attempt to reduce the appeal and uptake of these products among youth.^9^

There is a dearth of research on the inclusion of these synthetic cooling compounds in e-cigarette products available on the Australian market. Research has previously identified WS-3 and WS-23 in e-cigarette products available in the United States and Europe.^6,10–13^ A recent article by Jabba, et al.^10^ found that WS-3 was a common addition in e-liquid refills while WS-23 was commonly found in PuffBar branded disposable e-cigarettes in the United States. Similarly, Omaiye, et al.^12^ found that synthetic coolants were common in newer disposable devices compared to e-cigarette products purchased in the United States between 2016 and 2019. Since disposable e-cigarettes are emerging as a popular device type among youth in Australia^14^ it is essential to investigate if similar trends are identified in products sold on the Australian market.

## Methods

This study examined the content of bottled e-liquids, *N* = 33 (brands: Air Factory, Aqua, Bam Bam’s, Boardroom, Burst, Cloud Nurdz, Hi Drip, HILIQ, Juice Head, Liquid Nicotine Wholesalers, Salt Bae, and VaporFi) and disposable e-cigarettes, *N* = 37 (brands: Switch, HQD, Gunpod, IGET, PuffBar, Xtra, Trip, and Vaporlax) either purchased from Australian retailers, purchased online from stores which ship to Australia, or donated by Australian e-cigarette users. The e-liquids E1–E24 and disposables D1–D6 were purchased online from Liquid Nicotine Wholesalers in January 2021. The disposables D7 and D8 were purchased from CTC Bulli (Wollongong, Australia) in September 2021. The e-liquids E25–E29 were purchased from VaporFi in January 2020. The e-liquids E30–E33 and disposables D9–D38 were donated anonymously to the University of Wollongong in 2021 with purchased origin provided when specified by donor. For sample details see Tables 1 and 2.

**Table 1.** Concentration of Nicotine, WS-23, WS-3, and Menthol in Disposable E-cigarette Samples (D1–D37) in mg/mL (mean ± SD of Three Replicates)

Code	Brand and flavour name	Source	Origin	Nicotine form	Concentration (mg/mL)			
					Nicotine	WS-23	WS-3	Menthol
D1	Switch Blue Razz	Purchased	online (int)	Salt	34 ± 2	11.5 ± 0.3	—	1.96 ± 0.08
D2	Switch Cucumber Lime	Purchased	online (int)	Salt	35 ± 1	11.8 ± 0.4	—	2.5 ± 0.2
D3	Switch Grape	Purchased	online (int)	Salt	34.1 ± 0.7	11.4 ± 0.4	—	3.2 ± 0.1
D4	Switch POG	Purchased	online (int)	Salt	34 ± 1	11.2 ± 0.1	—	2.71 ± 0.03
D5	Trip Jungle Juice	Purchased	online (int)	Salt	34.1 ± 0.2	13.45 ± 0.0 7	—	—
D6	Xtra Mango Tango	Purchased	online (int)	Salt	34.8 ± 0.6	13.0 ± 0.2	—	—
D7	Gunnpod Lychee Ice	Purchased	shop	Salt	34.8 ± 0.4	32.7 ± 0.2	—	—
D8	HQD Curve Plus Lychee Ice	Purchased	shop	Salt	32.98 ± 0.08	18.9 ± 0.6	—	—
D9	HQD Blueberry	Donated	unknown	Salt	38 ± 2	19.6 ± 0.5	—	0.316 ± 0.009
D10	HQD Grapey	Donated	unknown	Salt	38 ± 1	13.0 ± 0.2	—	0.287 ± 0.002
D11	HQD Lemon Cake	Donated	unknown	Salt	31 ± 1	15.6 ± 0.5	—	—
D12	HQD Strawberry	Donated	unknown	Salt	40 ± 2	16.2 ± 0.8	—	—
D13	IGET Apple	Donated	unknown	Salt	38 ± 2	23.9 ± 0.8	—	—
D14	IGET Bubble Gum	Donated	unknown	Salt	37 ± 1	18.5 ± 0.5	1.21 ± 0.06	<LOQ
D15	IGET Cool Mint	Donated	unknown	Salt	37 ± 2	32 ± 2	—	4.0 ± 0.3
D16	IGET Grape	Donated	unknown	Salt	35.3 ± 0.6	15.3 ± 0.2	—	—
D17	IGET Grapefruit	Donated	unknown	Salt	36.1 ± 0.8	13.4 ± 0.6	—	—
D18	IGET Guava Ice	Donated	unknown	Salt	39 ± 1	16.0 ± 0.4	—	—
D19	IGET Guava Ice	Donated	unknown	Salt	39 ± 3	17.3 ± 0.4	—	—
D20	IGET Lychee Ice	Donated	unknown	Salt	37 ± 1	13.3 ± 0.5	—	—
D21	IGET Mango	Donated	unknown	Salt	37.5 ± 0.7	22.3 ± 0.4	—	—
D22	IGET Passion Fruit Mango	Donated	unknown	Salt	41.8 ± 0.5	30.4 ± 0.3	2.81 ± 0.03	—
D23	IGET Peach Ice	Donated	unknown	Salt	37 ± 1	24.2 ± 0.3		—
D24	IGET Pineapple Ice	Donated	unknown	Salt	37 ± 1	14.5 ± 0.1	—	<LOQ
D25	IGET Strawberry	Donated	unknown	Salt	37.7 ± 0.7	21.3 ± 0.2	—	-
D26	Puff Bar Blue Razz	Donated	unknown	Salt	37 ± 2	12.6 ± 0.1	—	<LOQ
D27*	Puff Bar Cool Mint	Donated	unknown	Salt	26 ± 1	8.8 ± 0.3	<LOQ	—
D28	Puff Bar Lychee Ice	Donated	unknown	Salt	36 ± 1	12.1 ± 0.1	—	—
D29	Puff Bar Lychee Ice	Donated	unknown	Salt	36.0 ± 0.9	19.5 ± 0.3	—	—
D30	Puff Bar O.M.G	Donated	unknown	Salt	38 ± 3	3.6 ± 0.2	—	—
D31**	Puff Bar Pomegranate	Donated	unknown	Salt	37.8 ± 0.7	10.5 ± 0.1	—	—
D32	Vaporlax Iced Cola	Donated	unknown	Salt	38 ± 2	37 ± 2	—	—
D33	Vaporlax Lush Ice	Donated	unknown	Salt	37 ± 1	4.5 ± 0.2	—	—
D34	Vaporlax Lush Ice	Donated	unknown	Salt	33.8 ± 0.7	4.46 ± 0.05	—	—
D35	Vaporlax Lush Ice	Donated	unknown	Salt	36 ± 1	5.4 ± 0.2	—	—
D36	Vaporlax Mango Ice	Donated	unknown	Salt	34 ± 1	21.2 ± 0.4	—	—
D37	Vaporlax Pink Lemonade	Donated	unknown	Salt	38.1 ± 0.4	16.4 ± 0.5	—	—

“Shop”: purchased for an Australian retail shop, “online (dom)” and “online (int)”: purchased from domestic online or international online providers that ship to Australia, “unknown”: data not collected for donated devices. * PuffBar Cool Mint; ** PuffBar Pomegranate; “—” below limit of detection (Nicotine 0.350 mg/mL, WS-23 0.146 mg/mL, WS-3 0.128 mg/ml, menthol 0.067 mg/mL) and “< LOQ” below limit of quantification (Nicotine 1.062 mg/mL, WS-23 0.441 mg/mL, WS-3 0.389 mg/mL, menthol 0.203 mg/mL).

**Table 2.** Concentration of Nicotine, WS-23, WS-3, and Menthol in E-cigarette Refill Liquids (E1 – E33) in mg/mL (mean ± SD of Three Replicates)

Code	Brand and flavor name	Purchased	Origin	Nicotine form	Concentration (mg/mL)			
					Nicotine	WS-23	WS-3	Menthol
E1	Air Factory Custard Craze	Purchased	online (int)	Free-base	6.76 ± 0.09	—	—	—
E2	Air Factory Iced Chee	Purchased	online (int)	Salt	37 ± 1	7.36 ± 0.07	—	1.17 ± 0.03
E3	Air Factory Mystery	Purchased	online (int)	Salt	31.3 ± 0.4	—	—	—
E4	Aqua Original Flow	Purchased	online (int)	Free-base	6.45 ± 0.09	—	—	—
E5	Bam Bam’s Original Cannoli	Purchased	online (int)	Free-base	3.36 ± 0.04	—	—	—
E6	Burst Melon Burst	Purchased	online (int)	Free-base	3.09 ± 0.02	—	—	—
E7	Burst Sher Burst	Purchased	online (int)	Free-base	6.7 ± 0.3	—	—	—
E8	Cloud Nurdz Salt Grape Apple	Purchased	online (int)	Salt	21.6 ± 0.2	—	—	—
E9	Cloud Nurdz Salt Strawberry Lemon	Purchased	online (int)	Salt	22.3 ± 0.1	—	—	—
E10	Hi Drip Mango Peach Iced	Purchased	online (int)	Free-base	3.1 ± 0.1	4.8 ± 0.2	—	—
E11	HILIQ Regular Tobacco	Purchased	online (int)	Salt	17.2 ± 0.6	—	—	—
E12	Juice Head Freeze Pineapple Grapefruit	Purchased	online (int)	—	—	4.5 ± 0.2	—	—
E13	Juice Head Pineapple Grapefruit SALT	Purchased	online (int)	Salt	25.6 ± 0.5	—	—	—
E14	Liquid Nicotine Wholesalers 99 problems	Purchased	online (int)	Free-base	14.4 ± 0.1	—	—	—
E15	Liquid Nicotine Wholesalers Bomb Wire	Purchased	online int)	Free-base	13.68 ± 0.08	—	—	—
E16	Liquid Nicotine Wholesalers Dynamite	Purchased	online (int)	Free-base	14.1 ± 0.3	—	—	—
E17	Liquid Nicotine Wholesalers Fair Hair	Purchased	online (int)	Free-base	13.5 ± 0.2	—	—	—
E18	Liquid Nicotine Wholesalers Nectar of the Gods	Purchased	online (int)	Free-base	14.7 ± 0.4	—	—	—
E19	Liquid Nicotine Wholesalers Strawberry Milk	Purchased	online (int)	Free-base	13.98 ± 0.08	—	—	—
E20	Liquid Nicotine Wholesalers Tigers blood	Purchased	online (int)	Free-base	14.2 ± 0.3	—	—	—
E21	Liquid Nicotine Wholesalers Vanilla Custard	Purchased	online (int)	Free-base	18.1 ± 0.3	—	—	—
E22	Naked 100 Salt Amazing Mango	Purchased	online (int)	Salt	32.2 ± 0.4	—	—	—
E23	Salt Bae Green Apple	Purchased	online (int)	Salt	25.1 ± 0.4	—	—	—
E24	Salt Bae Red Mango	Purchased	online (int)	Salt	26.2 ± 0.3	—	—	—
E25	VaporFi Classic Tobacco	Purchased	online (dom)	Free-base	12.6 ± 0.2	—	—	—
E26	VaporFi Dulce Leche	Purchased	online (dom)	Free-base	12.7 ± 0.3	—	—	—
E27	VaporFi Menthol Freeze	Purchased	online (dom)	Free-base	13.1 ± 0.2	—	—	19.1 ± 0.3
E28	VaporFi Strawberry	Purchased	online (dom)	Free-base	12.4 ± 0.2	—	—	—
E29	VaporFi Very Vanilla	Purchased	online (dom)	Free-base	12.8 ± 0.1	—	—	—
E30	Boardroom Vapor Apple Pie Moonshine	Donated	online (int)	Free-base	2.8 ± 0.1	—	—	—
E31	Boardroom Vapor Blackberry Vanillin Brandy	Donated	online (int)	Free-base	2.89 ± 0.03	—	—	—
E32	Boardroom Vapor Butterscotch	Donated	online (int)	Free-base	2.74 ± 0.05	—	—	—
E33	Boardroom Vapor Peanut Brittle	Donated	online (int)	Free-base	2.76 ± 0.09	—	—	—

“Shop”: purchased for an Australian retail shop, “online (dom)” and “online (int)”: purchased from domestic online or international online providers that ship to Australia, “unknown”: data not collected for donated devices. * PuffBar Cool Mint; ** PuffBar Pomegranate; “—” below limit of detection (Nicotine 0.350 mg/mL, WS-23 0.146 mg/mL, WS-3 0.128 mg/ml, menthol 0.067 mg/mL) and “< LOQ” below limit of quantification (Nicotine 1.062 mg/mL, WS-23 0.441 mg/mL, WS-3 0.389 mg/mL, menthol 0.203 mg/mL).

### Gas Chromatography-Mass Spectrometry (GC-MS)

Mass spectra were obtained using a Shimadzu QP2020 GC-MS single quadrupole system with electron ionization (Japan). Samples were introduced onto an SH-Rxi-5Sil MS capillary column (30 m × 0.25 mm × 0.25 µm) by injecting 1 µL of solution. The final method had a total run time of 13.67 minutes with a solvent cut time of 1.8 minutes. The injection temperature was 270°C with a split ratio of 1:10. The oven had an initial temperature of 60°C and was increased to 250°C at a rate of 15°C/min and held for 1 minute. Helium was used as the carrier gas at a flow rate of 0.95 mL/min. The mass spectrometer ion source temperature was 250°C and the interface temperature was set to 270°C. The mass spectra were obtained in scan mode over the 50–1000 *m/z* range.

Quantification of nicotine, menthol, and coolant molecules within e-liquids and disposables was achieved through creation of internal calibration curves using quinoline (Thermofisher Scientific Australia, North Ryde) as the internal standard (25 µg/mL). Analytical standards (≥98% purity) of WS-3 (*N*-ethyl-p-menthane-3-carboxamide), nicotine, and menthol were purchased from Sigma Aldrich (Castle Hill, Australia) and WS-23 (2-isopropyl-*N*,2,3-trimethylbutyramide) was purchased from Tokyo Chemical Industry (Chuo City, Japan). Individual stock solutions of each quantified compound were prepared in methanol (Thermofisher Scientific Australia, North Ryde) at 100 mg/mL. Volumes of these 100 mg/ml stock solutions were combined to obtain a mixed stock from which the working standards were obtained. Calibration curve concentration ranges were selected to prevent detector saturation and were as follows: Nicotine: 8–400 µg/mL, WS-23: 5–250 µg/mL, WS-3: 4–200 µg/mL, and menthol: 4–200 µg/mL. Six working standards were then analyzed by GC-MS to create the respective calibration curves.

### E-liquid Sample Preparation

The dilution factors for the e-liquids were selected to prevent saturation of the GC-MS detector during analysis and optimize the peak intensities for each flavoring molecule. The e-liquids (*N* = 33) and disposable samples (*N* = 37) were all diluted 1:200 (*v/v*) in methanol and spiked with quinoline (internal standard) at 25 µg/mL before analysis. All samples were prepared and analyzed in triplicate.

## Results

WS-23 was detected in every disposable e-cigarette sample analyzed in this study (Table 1) with concentrations ranging between 3.6 ± 0.2 (D30, Puff Bar O.M.G) and 37 ± 2 mg/mL (D32, Vaporlax Iced Cola) with a mean of 16.4 mg/mL. WS-3 was identified in only three disposable samples (D14, D22, and D27, 8%), all at a relatively low concentration (<3 mg/mL) compared to WS-23. Menthol was present in 27% of disposable e-cigarette samples (*n* = 10) with a mean concentration of 2.1 mg/mL, although only two samples (5%) were named as mint flavored (D15 and D27). Interestingly, D27 (“Puff Bar Cool Mint”) contained no detectable menthol despite its description as a “mint”-flavored e-liquid. Even though all the disposable devices contained cooling agents, only 38% of samples contained a cooling term in the flavor name (eg, “Iced Lychee”).

For the bottled e-cigarette liquid samples analyzed in this study, WS-3 was not detected in any samples and WS-23 was only detected in three samples (4.5 ± 0.2 to 7.36 ± 0.07 mg/mL; mean 5.6 mg/mL). The liquids which contained WS-23 were all fruit flavored and included a cooling term such as “iced” or “freeze” on the label.

Nicotine was also present in all disposable devices analyzed, with concentrations ranging from 26 ± 1 mg/mL (D27) to 41.8 ± 0.5 mg/mL (D22) and a mean concentration of 36.2 mg/mL (Table 1). The nicotine concentration of the bottled e-liquids was significantly lower than in the disposable devices (*p* < .001), with concentrations ranging from 2.74 ± 0.05 mg/mL (E32) to 37 ± 1 mg/mL (E2) and a mean concentration of 14.7 mg/mL (Table 2). In line with this finding, the bottled e-liquids contained mostly free-base nicotine samples, in contrast with the disposable devices, which were nicotine salts.

## Discussion

Our study determined the concentration of nicotine, two synthetic cooling agents (WS-3 and WS-23), and menthol in a range of disposable e-cigarettes and e-liquids available to Australian consumers. These synthetic cooling agents have been found to be increasingly used in the newer disposable e-cigarettes in the United States with high concentrations often observed.^10,12^ Australian disposable e-cigarettes were all found to contain WS-23 in concentrations comparable to those reported by Jabba, et al.^10^ and Omaiye, et al.^12^ Overall, use of WS-3 was uncommon (4%, *N* = 70) and only detected in disposable e-cigarettes, while WS-23 was more common in the Australian disposable e-cigarettes (100%, *N* = 37) than in bottled e-liquids (9%, *N* = 33).

Some differences were observed between this study and previous U.S. studies.^10,12^ The PuffBar Cool Mint disposable (D27, Table 1), analyzed in all three studies, had a lower concentration of WS-23 in this study. Interestingly, menthol was not detected in D27 in this study and WS-3 was detected in a much lower concentration compared to the previous studies. Most of the samples analyzed in this study were donated as used devices (Australian users, Wollongong area) which may have affected the concentration of some analytes in the residual liquids, particularly volatile molecules. As a result of this, it is likely that the more volatile compounds, including menthol, have been preferentially vaporized leaving little to no concentration of these volatile compounds at the end of the device life. The concentration of menthol measured may have been different in a new device of the same brand and flavor but was not analyzed here for comparison. The Puffbar Pomegranate disposable (D31, Table 1) was found to have a WS-23 concentration of 10.5 ± 0.1 mg/mL in our study. Jabba, et al.^10^ did not detect any WS-23 in a similarly labeled U.S. sample while Omaiye, et al.^12^ detected WS-23 at a lower concentration.

Despite no apparent correlations between nicotine concentration and presence of WS-23 in e-liquid refills, the addition of WS-23 in disposable cigarettes is more widespread than in e-liquid refill solutions. The inclusion of WS-23, in disposable e-cigarettes which all contained high concentrations of nicotine, may be an attempt to reduce the unpleasant throat irritation experienced by vapers upon inhalation of the high nicotine concentrations identified in these devices or allow tolerance to more intense flavors.^8,10^ This is particularly relevant in samples where there is no reference to “cool,” “iced” or “freeze” terminology on the label, where high concentrations of nicotine and WS-23 were still detected. The recent increase in the use of these disposable e-cigarette devices has been noted among nonsmoking youth who may be unaccustomed to the irritation caused by nicotine.^14^ Thus, it is possible that the inclusion of cooling agents may facilitate the uptake of e-cigarettes among youth.

The similarity between results from the United States and Australia demonstrates that trends in disposable e-liquid composition are comparable between the two countries, likely due to the same manufacturing sources. This study has also confirmed that this trend can be seen in a large range of e-cigarette brands and is not unique to PuffBar disposables. While WS-23 has not been included in the current bans of ingredients by the Australian government, it is concerning that concentrations observed here, are similar to those observed by Jabba, et al.^10^ that have been identified as unsafe based on thresholds recommended by the Joint FAO/WHO Expert Committee on Food Additives (JECFA).^15^ Cytotoxicity studies by Omaiye, et al.^12^ found that concentrations of WS-23 below those observed in e-liquids produced adverse effects in cell viability assays. Despite the fact that WS-23 is the main coolant used in vaping products identified in this study, it was not always the most commonly used coolant with WS-3 use reported in products from the United States and EU as recently as 2019.^6^ Considering the rapidly changing market and the wide range of other synthetic coolants available, which are considered by the Flavor and Extract Manufacturers Association of the United States (FEMA) as generally recognized as safe, there is potential for further alteration in the composition of these products. Further research is required to determine the potential inhalational toxicity of e-cigarette coolants WS-3 and WS-23 along with any additional coolants identified to evaluate the potential risks these pose to e-cigarette users.

## Supplementary Material

A Contributorship Form detailing each author’s specific involvement with this content, as well as any supplementary data, are available online at https://academic.oup.com/ntr.
